# Management of imatinib-associated skin rash in a patient with metastatic gastrointestinal stromal tumor: a case report

**DOI:** 10.1186/2045-3329-2-23

**Published:** 2012-12-04

**Authors:** Jean-Yves Blay

**Affiliations:** 1 Department of Medical Oncology, Centre Léon-Bérard, 28 rue Laennecx, Lyon28 rue Laennec, Lyon 69008, France

**Keywords:** Gastrointestinal stromal tumor, GIST, Metastasis, Imatinib mesylate, Skin rash

## Abstract

**Purpose:**

Long-term continuous imatinib is recommended for adult patients with unresectable and/or metastatic KIT+ gastrointestinal stromal tumors (GIST) as long as the patient continues to benefit. In the adjuvant setting, recent findings indicate that patients at considerable risk of recurrence should receive at least 3 years of imatinib. Because imatinib is often administered for prolonged periods, proper management of imatinib-associated adverse events is crucial.

**Case report:**

We report a 56-year-old man with metastatic KIT+ GIST of the liver who had Grade 3 imatinib intolerance (skin rash) when treatment was started. The rash was managed with antihistamine treatment (Dexchlorpheniramine maleate 4 mg per day) and several temporary (up to 2 weeks) dose interruptions. The patient’s skin rash partially improved, and he tolerated gradual reintroduction of imatinib over several months. The patient maintained imatinib 400 mg/d, and tolerated it during the 2 years when he was on antihistamine treatment. After 2 years, the patient continued imatinib therapy without having to take antihistamines. The patient responded according to RECIST 1.1 and Choi to imatinib treatment for his metastatic GIST (partial response). As of September, 2012, the patient has been on imatinib therapy for 131 months and remains progression free.

**Conclusions:**

The results of this case report demonstrated that a patient with metastatic KIT+ GIST who was initially intolerant to imatinib maintained, and responded to imatinib therapy after treatment of an imatinib-associated adverse effect. These results suggest that initial intolerance to imatinib should not necessarily result in treatment discontinuation, as these adverse effects, when managed properly, may be tolerated and may decrease over time.

## Background

Imatinib mesylate is approved for the treatment of adult patients with unresectable and/or metastatic KIT+ gastrointestinal stromal tumor (GIST) and for the adjuvant treatment of adult patients following resection of primary GIST [1]. Current treatment guidelines recommend long-term continuous imatinib therapy in patients with advanced GIST and at least 12 months of adjuvant imatinib therapy for patients with a substantial risk of relapse [2,3]. Recently reported results of the Scandinavian Sarcoma Group/Sarcoma Group of the Arbeitsgemeinschaft Internistische Onkologie (SSGXVIII/AIO) trial indicate that at least 3 years of adjuvant imatinib should be recommended in patients who have a considerable risk of recurrence after surgery [4]. National Comprehensive Cancer Network and ESMO Guidelines have recently been updated, based on the results of the SSGXVIII/AIO trial, endorsing this recommendation [5,6]. Because imatinib is often administered for prolonged periods of time, close monitoring and proper management of imatinib-associated adverse effects are crucial. Imatinib is generally well tolerated, although some patients have difficulty tolerating the standard starting dose of 400 mg/d. Here, we report on a patient with metastatic GIST who experienced skin toxicity in response to imatinib at the beginning of standard therapy. With prudent use of supportive care measures, the patient was able to continue imatinib therapy while remaining progression free for more than 10 years.

## Case presentation

**Figure 1:**
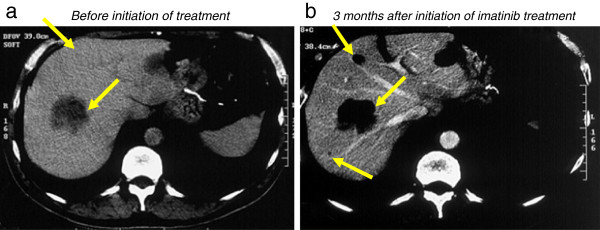
**CT scans of the patient before and after imatinib treatment for metastatic GIST. **Tumor masses (arrow) can be seen in the liver of the patient before initiation of imatinib treatment (**a**). The masses (arrow) became hypodense and homogenous on CT obtained 3 months after imatinib treatment (**b**), indicating good response to imatinib.

A 56-year-old man with a disease diagnosis of primary gastric GIST underwent complete en-bloc resection in 1998. Mutational analysis of the excised mass showed that the tumor had a mutation in *KIT* exon 11 involving codons 557 and 558. Based on the tumor size (7 cm) and mitotic index (> 8 mitoses per 50 high-powered field), the patient was considered at high risk for recurrence [7]. In 2000, the patient developed metastatic GIST, and computed tomography (CT) scans revealed at least 8 large masses in both liver lobes with the largest liver mass measuring 8 cm. He was started on imatinib 400 mg/d in April 2001, but developed Grade 3 skin rash after 4 weeks of imatinib therapy. The rash was managed with antihistamine treatment (dexchlorpheniramine 4mg per day and oxatomide 60 mg per day) and several temporary dose interruptions (each dose interruption lasted for a few days to up to 2 weeks). After each dose interruption, the patient was restarted on imatinib 200 mg/d, then after 1 week, 300 mg/d, and resumed taking standard-dose imatinib 400 mg/d after 9 weeks. The patient’s skin rash partially improved in response to dexchlorpheniramine treatment and dose interruption, and he maintained and tolerated imatinib 400 mg/d during the 2 years when he was on antihistamine treatment. After 2 years, although his skin rash was not completely resolved, the patient was able to continue imatinib therapy without having to take antihistamines. The patient responded to imatinib treatment for his metastatic GIST in the liver. Tumors became hypodense after 3 months of imatinib therapy (Figure 1), indicating good response to imatinib according to Choi criteria and Response Evaluation Criteria In Solid Tumors (RECIST). After 122 months of treatment, a partial response by RCIST and Choi criteria is still observed; liver masses had decreased in size from 8 to 5 cm. As of September, 2012, the patient has been on imatinib therapy for more than 10 years and remains progression free with normal physical examination.

This case report demonstrates that a patient with metastatic GIST who has difficulty tolerating imatinib at the standard treatment initiation dose (400 mg/d) can be effectively managed with supportive care and dose adjustments in order to maintain response to treatment. This suggests that initial intolerance to imatinib should not necessarily result in treatment discontinuation, as some of these adverse effects, when managed properly, can be tolerated and may decrease over time.

Maintaining continuous drug administration at a sufficiently high dose is necessary for GIST patients to obtain clinical benefit from imatinib [8,9]. However, adverse effects may reduce patient compliance and the clinical efficacy of the treatment. Although true imatinib intolerance is very rare, short-term intolerance and discomfort are not uncommon [10]. The most frequently observed adverse effects, generally Grade 1 or 2, include edema, gastrointestinal effects (diarrhea or nausea/vomiting), skin rash, and fatigue. There are different strategies for managing each imatinib-related adverse effect [10]. Skin rash, for example, which often occurs during the first few months of treatment initiation, can be managed with antihistamines and topical steroids [10]. In patients with severe skin reactions, dose reduction or interruption of imatinib, and/or use of oral steroids may be required [10]. Imatinib can be gradually reintroduced, and many patients, like the patient described in this case report, are eventually able to tolerate imatinib.

This patient has continued treatment with imatinib and has remained disease free for more than 10 years. This is one of the longest durations of imatinib treatment for advanced KIT+ GIST reported in the literature. Long-term follow-up results of a key trial in patients with metastatic/advanced GIST (B2222 study) recently showed that 18% of the 147 patients initially enrolled in the study remained on continuous imatinib at a median follow-up time of 9.4 years (maximum 9.9 years) [11]. The results of this case report and the B2222 study support the long-term efficacy of imatinib for the treatment of patients with unresectable and/or metastatic KIT+ GIST.

## Conclusions

In conclusion, early recognition and proper management of imatinib-associated adverse effects may help GIST patients maintain imatinib therapy and ultimately achieve optimal clinical efficacy.

## Consent

Written informed consent was obtained from the patient for publication of this case report and any accompanying images. A copy of the written consent is available for review by the Editor-in-Chief of this journal.

## Abbreviations

CT: Computed tomography; GIST: Gastrointestinal stromal tumor; SSGXVIII/AIO: Scandinavian Sarcoma Group/Sarcoma Group of the Arbeitsgemeinschaft Internistische Onkologie.

## Competing interests

Jean-Yves Blay received research grants and honoraria from Novartis, Pfizer, GlaxoSmithKline, Roche, and PharmaMar.

## Authors’ contributions

JYB managed the patient in the clinic, collected and analyzed data, critically revised each draft of the manuscript, and approved the final version.
